# A Lightweight Image Encryption Algorithm Based on Chaotic Map and Random Substitution

**DOI:** 10.3390/e24101344

**Published:** 2022-09-23

**Authors:** Yousef Alghamdi, Arslan Munir, Jawad Ahmad

**Affiliations:** 1 Department of Computer Science, Kansas State University, Manhattan, KS 66506, USA; 2 School of Computing, Edinburgh Napier University, Edinburgh EH10 5DT, UK

**Keywords:** image encryption, logistic map, chaotic system, S-box, permutation, substitution

## Abstract

Chaotic-maps-based image encryption methods have been a topic of research interest for a decade. However, most of the proposed methods suffer from slow encryption time or compromise on the security of the encryption to achieve faster encryption. This paper proposes a lightweight, secure, and efficient image encryption algorithm based on logistic map, permutations, and AES S-box. In the proposed algorithm, SHA-2 based on the plaintext image, a pre-shared key, and an initialization vector (IV) are used to generate the initial parameters for the logistic map. The logistic map chaotically generates random numbers, which are then used for the permutations and substitutions. The security, quality, and efficiency of the proposed algorithm are tested and analyzed using a number of metrics, such as correlation coefficient, chi-square, entropy, mean square error, mean absolute error, peak signal-to-noise ratio, maximum deviation, irregular deviation, deviation from uniform histogram, number of pixel change rate, unified average changing intensity, resistance to noise and data loss attacks, homogeneity, contrast, energy, and key space and key sensitivity analysis. Experimental results reveal that the proposed algorithm is up to 15.33× faster compared to other contemporary encryption methods.

## 1. Introduction

Information security has an important role when it comes to sharing data. Many cryptography algorithms have been proposed for the secure storage of information on computer systems, as well as secure transfer of information over a network. Digital images are one form of sensitive data that need to be stored and transmitted securely [1]. Digital images are two-dimensional arrays with a certain number of channels (one for grayscale images, three for color images, and four for color images with a transparency channel) that store pixel values. Digital images tend to have a high redundancy due to correlation between neighboring pixels.

Image encryption relies on two techniques to create cipher images and reduce the correlation between neighboring pixels: confusion and diffusion [2]. Confusion is often achieved via substitution, which is the change of the values of each pixel in a digital image by a substitution map. In image encryption, confusion is controlled by using a key to obscure the plaintext image values [3]. Diffusion means that if a single pixel is changed in the plaintext image, then it should result in a change in about half of the pixels in the cipher image, and similarly, if a single pixel is changed in the cipher image, then it should result in a change in about half of the pixels in the plaintext image. Diffusion helps reduce the correlation between adjacent pixels in a plaintext image, which is accomplished by a permutation in the image encryption algorithms.

Research in image encryption relies on a set of parameters to evaluate the security and efficiency of an image encryption method [4]. Some of the parameters are used to quantify the diffusion characteristics of an image encryption method, such as mean square error (MSE), mean absolute error (MAE), number of pixel change rate (NPCR), and unified average change intensity (UACI). To measure the quality of the encryption, metrics such as chi-square, peak signal-to-noise ratio (PSNR), maximum deviation, irregular deviation, deviation from uniform histogram, and resistance to noise and data loss attacks are used. The correlation coefficient and local and global entropy are parameters that assess the security of the encryption method. When deciding on the specific order of confusion and diffusion steps while designing an encryption algorithm, it needs to be ensured that the steps are reversible so that the cipher image can be decrypted. Furthermore, computational time and energy consumption of the image encryption algorithm are additional factors to consider, depending on the application domain.

Most of the prior works on image encryption [5,6,7,8] do not consider encryption speed in their design. In this work, we propose a lightweight image encryption algorithm considering the encryption speed. In our proposed lightweight image encryption algorithm, chaotic maps are selected for their minimal computation requirement, low complexity, and fast speed in generating pseudorandom sequences. The algorithm utilizes a pre-shared symmetric key $k_{sym}$ and an initialization vector (IV) to randomly generate two 2D matrices that are used to perform the confusion and diffusion on the plaintext image. The pre-shared symmetric key $k_{sym}$ is XORed with a hash of the plaintext image to obtain a derived key. The IV is a generated random data block for each run of the algorithm so that encryption of the same plaintext image yields a totally different cipher image for each run of the algorithm.

Our main contributions in this article are as follows:

- Developing a lightweight image encryption algorithm without compromising the security, quality, and efficiency metrics.
- Evaluating the proposed image encryption algorithm with a comprehensive set of evaluation metrics, such as correlation coefficient, histogram and chi-square tests, local and global entropy analysis, encryption quality, diffusion characteristics, resistance to differential attacks, resistance to noise and cropping attacks, and key sensitivity. The evaluation results of our proposed algorithm are compared with existing image encryption algorithms.
- Utilizing an IV with the proposed algorithm so that encryption of the same plaintext image with the proposed algorithm yields totally different cipher images in each run of the algorithm, which helps in hiding statistical patterns in image encryption algorithms, thus making cryptanalysis difficult.
- Testing the randomness of the cipher images produced by the proposed image encryption algorithm using the National Institute of Standards and Technology (NIST) test suite.

The rest of the paper is organized as follows. Section 2 discusses image encryption algorithms in the literature. Section 3 presents the proposed image encryption algorithm along with its flowchart and pseudocode. Section 4 discusses the parameters used in this paper to evaluate the proposed algorithm, and also includes the detailed results and evaluation of the proposed algorithm. Finally, Section 5 concludes this work.

## 2. Related Work

Many image encryption techniques have been proposed in the literature. Hua et al. [5] have proposed an image encryption algorithm based on a 2D chaotic map, where the authors have used a 2D sine-logistic map to generate two matrices. The proposed algorithm uses one of the generated matrices to randomly shuffle the image pixel positions by connecting pixels in different rows and columns into circles, and shifting them within the circles. Then, the algorithm proceeds to do row and column substitutions on the pixel values of the resulting permuted image. The diffusion and confusion steps are repeated using the second matrix. The performance of the proposed method is fast, but it has a high correlation between the pixels of the encrypted image. The algorithm proposed by Alanezi et al. [6] utilizes two chaotic maps: a logistic-sine map is used to permute the plaintext image, and a logistic–Chebyshev map is used to substitute the resulting permuted image. The algorithm then performs an XOR operation on the substituted image with a cascading of the two maps to produce the cipher image.

The algorithm proposed by Arif et al. [7] is based on logistic maps. The proposed algorithm uses the plaintext image to generate a hash, which is then divided into four parts, each of which is used as an initial parameter input for the logistic maps to generate four pseudorandom number arrays. The algorithm then performs row and column permutations using the first and second keys, respectively. An XOR operation is performed on the resulting image using the third key. The last step is to perform a substitution on the image using either AES S-Box or AES revers S-Box based on the fourth generated key. However, the method proposed by Arif et al. does not perform efficiently in terms of encryption time. The encryption method proposed by Lu et al. [9] is based on Logistic-Sine maps, and uses a single S-Box. The proposed algorithm starts by generating the S-Box and chaotic sequence using pre-shared keys as input parameters. The chaotic sequence is used to permute the plaintext image. The resulting image is then substituted twice using the chaotic sequence as a key for the S-Box. The proposed logistic-sine system does not require the use of modular operation, which leads to a slight speed-up but is not that significant in the overall encryption time.

Wang et al. [8] have used Josephus traversing and a mix of four chaotic maps. The proposed algorithm has a three-round scrambling process using Josephus traversing and logistic map, and one round of diffusion using one of logistic, Chebyshev, sine, or cosine maps based on a mod operation on the pixels of the plain image. This proposed algorithm does not perform confusion on the pixel values. The algorithm proposed by Wang et al. has good security properties, as observed through evaluation metrics, but the encryption speed of the algorithm is slow.

In recent years, many compressive-sensing-based image encryption algorithms have been proposed. Compressive sensing is helpful in reducing the time and size of encrypted images by sampling, compressing, and encrypting the image at the same time [10]. Ye et al. [11] have proposed a new chaotic system that has a hyperchaotic behavior. The images are first compressed using compressive sensing, and the resulting compressed image is then encrypted using a public key elliptic curve encryption algorithm. The proposed algorithm can encrypt two images at the same time, reducing the encryption times of multiple images.

Although many prior works have proposed image encryption algorithms, most of the prior works focus only on security aspects of the proposed algorithms, and do not consider the encryption speed. Furthermore, prior works evaluate their proposed encryption algorithm with some security metrics; however, many of the prior works do not evaluate the proposed algorithms with a comprehensive set of metrics. Additionally, most of the prior works evaluate their proposed algorithm on grayscale images and not on color images, which are the dominant form of images in this era. This work fills the void in prior works by evaluating the proposed algorithm for both grayscale and color images. Furthermore, we evaluate the proposed algorithm with a comprehensive set of evaluation metrics for image encryption. Finally, we design the proposed algorithm considering the encryption speed so that the proposed algorithm can be deployed for real-time applications and devices with limited resources, such as Internet of things and edge devices.

## 3. Proposed Image Encryption Algorithm

**Figure 1:**
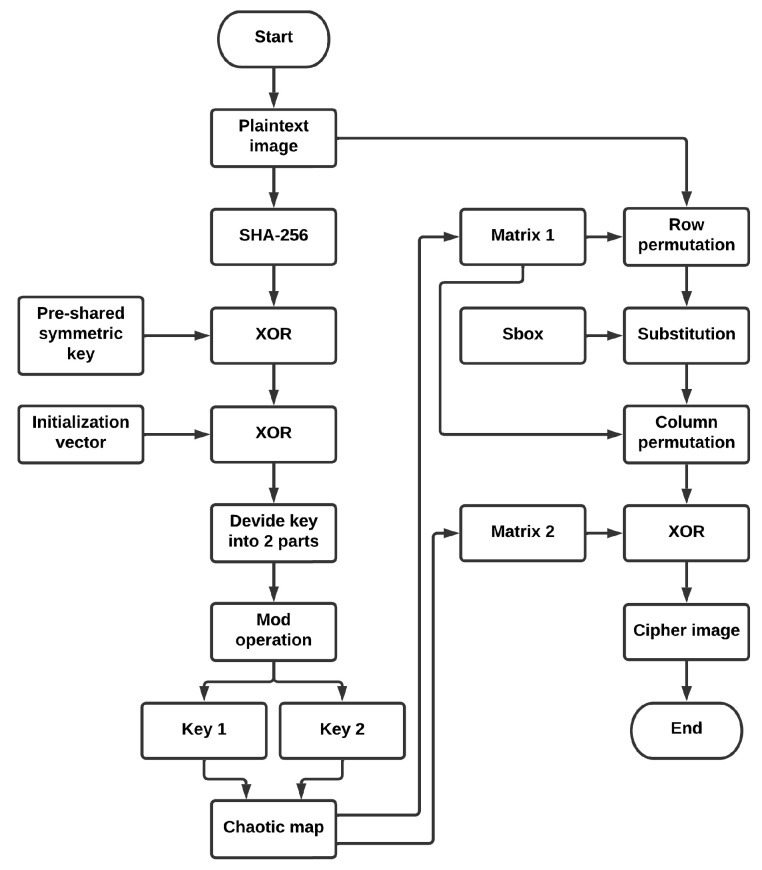
Proposed image encryption algorithm’s flowchart.

The flowchart for the proposed lightweight image encryption algorithm is shown in Figure 1. The algorithm is designed to be lightweight, and it performs faster than other image encryption methods in the literature without compromising the encryption quality and security metrics. The proposed method utilizes a logistic map to perform row and column permutation, and the AES S-Box and an XOR operation to perform the substitution. The AES S-Box is a non-linear substitution table that maps an 8-bit input to an 8-bit output [12].

**Figure 2:**
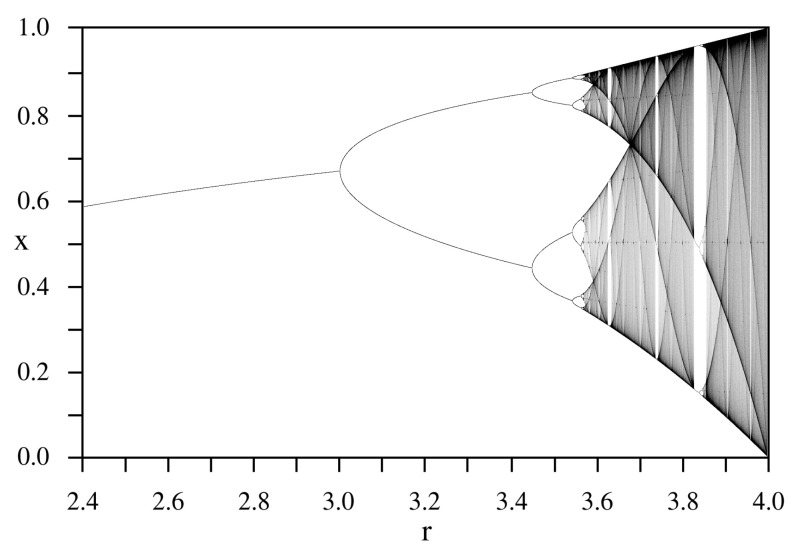
Logistic map bifurcation diagram.

The logistic map is used to generate two matrices that are utilized to perform confusion and diffusion on the plaintext image. The logistic map is given by the following recursive equation:

(1) $$X_{n+1}=rX_{n}\left(1-X_{n}\right),$$

where $X_{0}$ is the starting population or initial parameter, the *X* range is [0, 1], and *r* is the growth rate or control parameter, which has a range of [0, 4], but the map only start to behave chaotically when *r* is in the range [3.56995, 4] [13]. Figure 2 depicts the bifurcation diagram of the logistic map. The S-Box and XOR operation are used to obscure the plaintext image values and reduce the correlation between the image pixels.

The proposed algorithm is comprised of two main functions. The first function generates the encryption keys matrices, using a combination of a hash generated from the plain text image using SHA-2 256, a pre-shared secret key, and an IV that is generated randomly for each run of the algorithm. SHA-2 256 is a hashing function that was developed by the United States National Security Agency (NSA). Hashing is a mathematical function that transforms data of any size into a bit array with a fixed size [14]. Even though SHA-3 is newer and more secure than SHA-2 256, we have not used SHA-3 in the proposed algorithm because of its higher computational complexity of SHA-3 as compared to SHA-2. The hash generated from the plaintext image, pre-shared secret key, and IV are XORed to generate a 256-bit derived key, which is then split into two 128-bit parts (${\mathrm{Key}}_{1}$, ${\mathrm{Key}}_{2}$) that go through a mod 0.9999 operation to be suitable for use as an initial parameter for the logistic map. The main purpose of the IV is that the same plaintext image produces totally different cipher images on multiple runs of the algorithm even though the pre-shared key has not changed. Thus, IV helps in hiding the statistical pattern in image encryption, which makes cryptanalysis difficult. Key1 and Key2 are used to generate Matrix 1 ($M_{1}$) and Matrix 2 ($M_{2}$), respectively. The second function of the proposed algorithm is the confusion and diffusion transformation to encrypt the plaintext image. $M_{1}$ is used to chaotically permute the rows and the columns of the plaintext image. AES S-Box is used to obscure the plaintext image values, and $M_{2}$ is used in an XOR operation on the resulting permuted and substituted image.

Algorithm 1 depicts the pseudocode of the proposed image encryption algorithm. Table 1 shows a sample of the generated $M_{1}$ values. To illustrate the generation of the matrices, let us assume that the ${\mathrm{Key}}_{1}$ initial value is 0.78648 (line 5 in Algorithm 1). The first iteration of the matrix generation uses the initial ${\mathrm{Key}}_{1}$ value to calculate the new ${\mathrm{Key}}_{1}$ using the logistic map (Equation (1)). The new ${\mathrm{Key}}_{1}$ value comes out to be 3.99876×0.78648×(1−0.78648)=0.67151. The first value of $M_{1}$ is calculated using the new ${\mathrm{Key}}_{1}$ (line 10 in Algorithm 1) as $0.67151\times 10^{6}\;\mathrm{mod}\;256=671,510\;\mathrm{mod}\;256=22$. It should be noted that we round the results to five significant figures for the fractional part of numbers in our computations for $M_{1}$ and $M_{2}$. In the second iteration, ${\mathrm{Key}}_{1}$ from the previous iteration is used in the logistic map, and the new ${\mathrm{Key}}_{1}$ value comes out to be 3.99876×0.67151×(1−0.67151)=0.88206. The second value of $M_{1}$ is calculated using the new ${\mathrm{Key}}_{1}$ as $0.88206\times 10^{6}\;\mathrm{mod}\;256=882,060\;\mathrm{mod}\;256=140$. Similarly, other values of $M_{1}$ are calculated following the steps in Algorithm 1.

The steps of the proposed algorithm to encrypt an image are as follows:

1. Read plaintext image *P*.
2. Generate a hash from plaintext image *P* using SHA-2 256 (line 2 in Algorithm 1).
3. Generate the IV random data block.
4. Perform XOR operation between the image hash and the pre-shared secret key $k_{sym}$ (line 3 in Algorithm 1).
5. Perform XOR operation between the result of Step 4 and the IV (line 4 in Algorithm 1).
6. Divide the result of Step 5 into two equal 128-bit parts.
7. Map the two parts from Step 6 between 0 and 0.9999 (by converting the hexadecimal hash part into an integer and then taking modulus 0.9999) and save as keys ${\mathrm{Key}}_{1}$ and ${\mathrm{Key}}_{2}$ (lines 5 and 6 in Algorithm 1).
8. Use the two keys as an initial parameter for the chaotic map to generate Matrix 1 ($M_{1}$) and Matrix 2 ($M_{2}$) (lines 7–11 in Algorithm 1).
9. Use $M_{1}$ to perform chaotic row permutation on *P* (line 14 in Algorithm 1).
10. Use the AES S-Box to replace the pixel values of the resulting permuted image from Step 9 (line 15 in Algorithm 1).
11. Use $M_{1}$ to perform chaotic column permutation on the resulting substituted image from Step 10 (line 16 in Algorithm 1).
12. Perform XOR operation between the resulting permuted image from Step 11 and Matrix 2 ($M_{2}$) to produce the cipher image *C* (lines 17 and 18 in Algorithm 1).

**Algorithm 1:** Proposed lightweight image encryption.
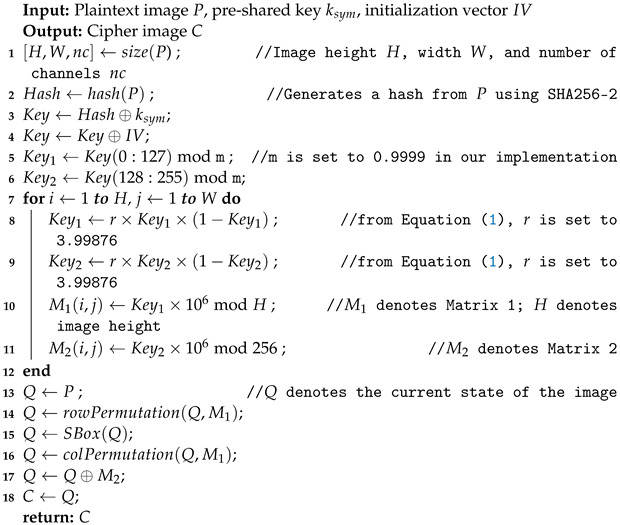


**Figure 3:**
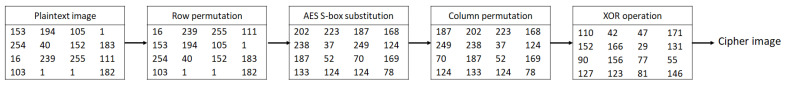
The proposed algorithm applied to a 4×4 sample.

To illustrate these steps further, the proposed algorithm is applied on 4×4 sample data, as presented in Figure 3. For decryption, the cipher image *C*, IV, and the hash of the image are sent to the receiver. The SHA-2 256 hash is securely sent to the receiver along with the key via a secure key exchange algorithm, such as Diffie–Hellman key exchange. The steps of the decryption process for the proposed algorithm are discussed below:

1. Read the cipher image *C* along with the SHA-2 256 hash and the IV.
2. Perform Steps 4 to Steps 8 of the encryption process.
3. Perform XOR operation between the cipher image *C* and $M_{2}$.
4. Use $M_{1}$ to perform chaotic column permutation on the resulting XORed image from Step 4.
5. Use the AES reverse S-Box to replace the pixel values of the resulting permuted image from Step 5.
6. Use $M_{1}$ to perform chaotic row permutation on the resulting substituted image from Step 6 to produce the decrypted image *I*.

## 4. Results and Analysis

**Figure 4:**
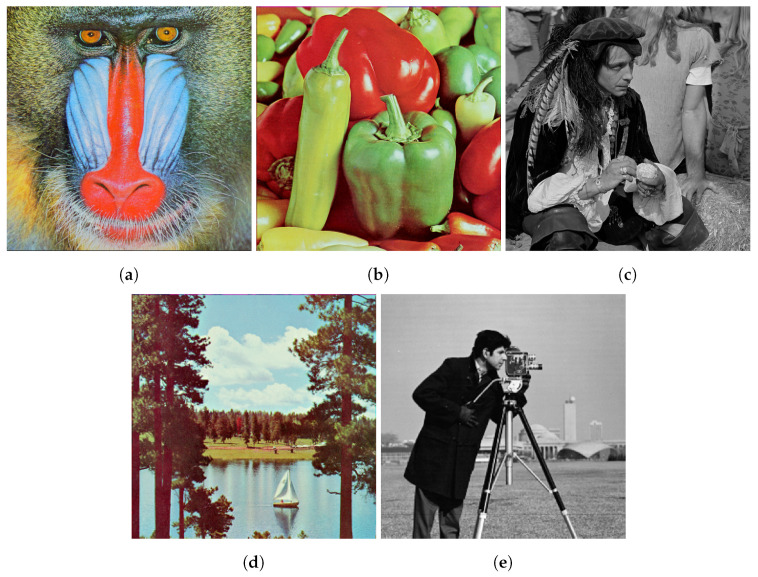
Images used to test the proposed algorithm: (**a**) Baboon, (**b**) Peppers, (**c**) Male, (**d**) Sailboat, and (**e**) Cameraman.

**Figure 5:**
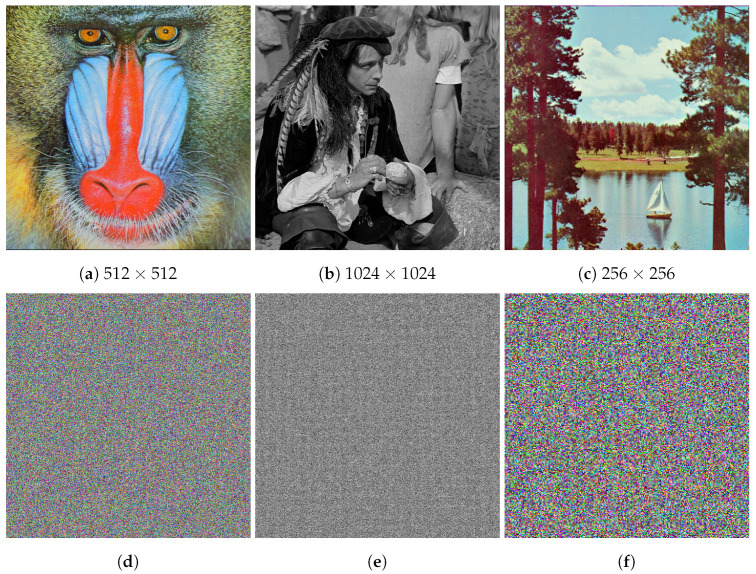
Sample images and their respective encrypted ciphers: (**a**) Baboon, (**b**) Male, (**c**) Sailboat, (**d**) Baboon cipher, (**e**) Male cipher, (**f**) Sailboat cipher.

To evaluate the proposed algorithm, we have used a set of standard test images found in the literature [15], namely Baboon, Peppers, Male, Sailboat, and Cameraman, as shown in Figure 4. The proposed algorithm is tested on 8-bit grayscale images and 24-bit color images with variable resolutions of 128×128, 256×256, 512×512, and 1024×1024 pixels. A set of plaintext images and their corresponding cipher images are shown in Figure 5. The proposed algorithm in this paper is analyzed and compared to methods from related literature for time complexity, correlation coefficient, analyses of histogram, chi-square, local and global entropy, encryption quality (MSE, MAE, PSNR, maximum deviation, irregular deviation, and deviation from uniform histogram), resistance to differential attacks, NPCR, UACI, resistance to noise and data loss attacks, homogeneity, contrast, energy, key space, and key sensitivity. The NIST SP 800-22 test suite is also used to test the randomness characteristics of the proposed algorithm. Each of these tests and their results are discussed in detail below.

### 4.1. Encryption Speed

The machine used to test the performance of the proposed algorithm has an Intel Core i5 CPU running at 3.5 GHz. The machine has 12 GB of RAM and runs the Windows 10 operating system. The proposed algorithm is run in MATLAB R2022b. Table 2 shows the results of execution time of the algorithm on images with sizes 256×256, 512×512, and 1024×1024. Table 3 compares the encryption speed of the proposed algorithm with the speeds of several related image encryption methods in the literature. The results indicate that the proposed algorithm has the fastest encryption speed. In Table 4, the execution times of the related encryption methods have been scaled to 3.5 GHz CPU to match with the processor frequency of the machine on which the proposed algorithm is run. Even though scaling does not provide 100% accuracy for processor runtime because of different instruction set architectures and memory subsystems, scaling provides reasonable estimates and facilitates relative comparisons [16]. Results indicate that the proposed algorithm still has the best encryption speed after scaling. The proposed algorithm has a speed increase of 2.36× compared to [6], 15.30× compared to [9], and up of 2.29× when compared to [17].

### 4.2. Correlation Coefficient Analysis

Images tend to have a high correlation between neighboring pixels. Image encryption methods aim to reduce this correlation to help obscure the image. The correlation values are in the range [1, −1], where 0 means no correlation between the two pixels, and 1 and −1 represent the maximum positive and negative correlation, respectively. For an image encryption algorithm to be considered secure, the correlation values should be as close to 0 as possible. To test the correlation of the pixels in the cipher image, two methods are used: (i) correlation coefficient between adjacent pixels of the cipher image [18], and (ii) correlation coefficient between the plaintext image and its cipher [19]. The correlation coefficient is calculated and compared for the horizontal, vertical, and diagonal pixels of the image.

#### 4.2.1. Correlation Coefficient of Adjacent Pixels

The vertical correlation can be calculated as:

(2) $$\begin{array}{c} CC_{v}=\frac{\sum_{i=1}^{H-1}\sum_{j=1}^{W}\left(C_{\left(i,j\right)}-\bar{C}\right)\left(C_{\left(i+1,j\right)}-\bar{C}\right)}{\sqrt{\sum_{i=1}^{H-1}\sum_{j=1}^{W}{\left(C_{\left(i,j\right)}-\bar{C}\right)}^{2}\sum_{i=1}^{H-1}\sum_{j=1}^{W}{\left(C_{\left(i+1,j\right)}-\bar{C}\right)}^{2}}}, \end{array}$$

where *H* and *W* are the height and width of the image, respectively; and $C_{\left(i,j\right)}$ and $C_{\left(i+1,j\right)}$ are adjacent pixel values of the cipher image at position (i,j) and position (i+1,j), respectively. $\bar{C}$ is the mean of the pixel values of the cipher image. To calculate the horizontal and the diagonal correlation coefficient, Equation (2) can be modified to use the pixel values at $C_{\left(i,j+1\right)}$ and $C_{\left(i+1,j+1\right)}$, respectively. The results for the vertical, horizontal, and diagonal correlation coefficients of adjacent pixels for the cipher images for the proposed algorithm are presented in Table 5. Table 6 compares vertical, horizontal, and diagonal correlation coefficients for the proposed algorithm to related encryption methods in the literature. The results show that the proposed algorithm has smaller values of correlation coefficients compared to other algorithms, but the performance of the encryption algorithm by Alanezi et al. [6] is better than the proposed algorithm for this metric, though the results of the proposed algorithm are still very close to that of the encryption algorithm by Alanezi et al. [6].

#### 4.2.2. Correlation Coefficient between Plaintext and Cipher Images

The mathematical expression of the correlation coefficient between plaintext and their cipher images can be calculated as:

(3) $$\begin{array}{c} CC_{P,C}=\frac{\sum_{i=1}^{H}\sum_{j=1}^{W}\left(P_{\left(i,j\right)}-\bar{P}\right)\left(C_{\left(i,j\right)}-\bar{C}\right)}{\sqrt{\sum_{i=1}^{H}\sum_{j=1}^{W}{\left(P_{\left(i,j\right)}-\bar{P}\right)}^{2}\sum_{i=1}^{H}\sum_{j=1}^{W}{\left(C_{\left(i,j\right)}-\bar{C}\right)}^{2}}}, \end{array}$$

where *H* and *W* are the height and width of the images, respectively. $P_{\left(i,j\right)}$ and $C_{\left(i,j\right)}$ are pixel values at index i,j of the plaintext image and cipher image, respectively. $\bar{P}$ and $\bar{C}$ are the mean of the pixels values of plaintext image and cipher image, respectively. The vertical, horizontal, and diagonal correlation coefficients of the proposed algorithm are presented in Table 7. Results show that the proposed algorithm has small correlation values between the plaintext and cipher images.

### 4.3. Histogram Analysis

**Figure 6:**
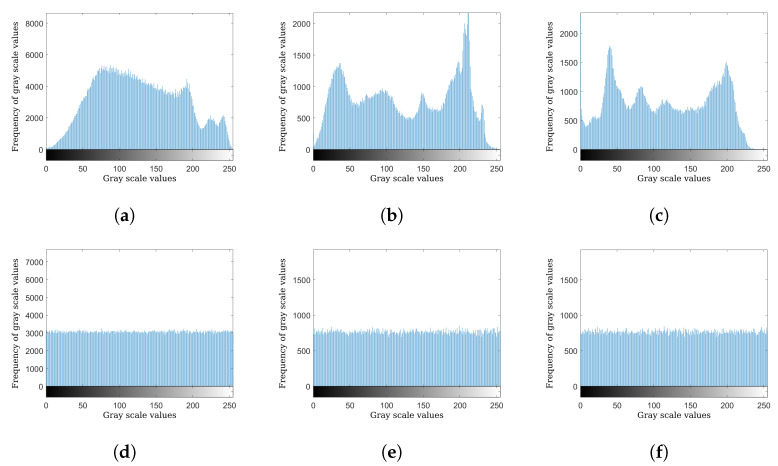
Histograms of images and their respective ciphers: (**a**) Baboon histogram, (**b**) Sailboat histogram, (**c**) Peppers histogram, (**d**) Baboon cipher histogram, (**e**) Sailboat cipher histogram, (**f**) Peppers cipher histogram.

The histogram of an image is used to illustrate the distribution of the values of an image’s pixels. In image encryption, histogram analysis is utilized to check the uniformity of the histogram of the cipher images. If a cipher image has a uniform histogram, then it is considered to be more secure against statistical attacks. Figure 6 shows the histograms of plaintext images Baboon, Sailboat, and Peppers, and their respective ciphers. The cipher images present uniformly distributed histograms, which illustrate the proposed algorithm’s efficiency in hiding the plaintext image’s information, and resilience towards histogram analysis attacks. To quantify the histogram analysis, chi-square ($\chi^{2}$) test can be used, which is mathematically represented as:

(4) $$\chi^{2}=\sum_{i=0}^{255}\frac{{\left(f_{i}-E\right)}^{2}}{E},$$

$$E=\frac{H\times W}{256},$$

where *f* represents the histogram of the cipher image, $f_{i}$ denotes the histogram value at index *i*, E denotes the expected value (mean) of the cipher image, and *H* and *W* signify the height and width of the image, respectively. The lower the value of $\chi^{2}$, the closer the distribution of the encrypted image is to the uniform distribution. A uniform histogram has a chi-square value of 0. Table 8 shows the chi-square test values for plaintext and cipher images encrypted by the proposed algorithm. Results in Table 8 indicate that the $\chi^{2}$ values of the cipher images encrypted by our proposed algorithm are extremely low, and thus the histograms of cipher images encrypted by our proposed encryption algorithm are very close to uniform distribution.

### 4.4. Entropy Analysis

Entropy is a statistical test that measures unpredictability and randomness, which was introduced by Claude Shannon in 1948 [20]. Entropy is used to quantify the uncertainty in communication systems. In image encryption, a cipher image that has a high entropy value obfuscates the plaintext better than cipher images with low entropy. Entropy is mathematically represented as:

(5) $$H\left(m\right)=-\sum_{i=0}^{2^{n}-1}p\left(m_{i}\right)\log_{2}\left[p\left(m_{i}\right)\right],$$

where *n* represents the number of bits used to represent the symbol $p(m_{i})$, and $p(m_{i})$ represents the probability of symbol $m_{i}$, that is, the probability of occurrence of intensity *i* for a pixel in the image. Entropy for an image is calculated using Equation (5), where $p(m_{i})$ represents normalized histogram counts for each intensity value in the image. Since the maximum possible intensity values for a pixel in the 8-bit pixel representation are 256, the ideal entropy for an image can be calculated using Equation (5) as follows:

$$H_{ideal}=-\sum_{i=0}^{255}\frac{1}{256}\times \log_{2}\frac{1}{256}=8$$

The entropy of a cipher image produced by an encryption algorithm should be as close as possible to the ideal entropy value of 8. Cipher images with low entropy values are weaker against brute force attacks. Shannon entropy is considered a *global entropy* because it measures the pixel information of the entire image. The *local entropy* is measured by computing the mean Shannon entropy of randomly selected non-overlapping blocks. Local entropy has better accuracy, consistency, and efficiency over global Shannon entropy [21]. Local entropy can be calculated as:

(6) $$H_{k,T_{B}}\left(S\right)=\sum_{i=1}^{K}\frac{H(S_{i})}{K},$$

where *S* is a set of randomly selected non-overlapping blocks containing $T_{B}$ pixels, and *K* is the number of random blocks. $H(S_{i})$ is the Shannon entropy (from Equation (5)) of the *i*th block. The results of the entropy analysis of the proposed algorithm are presented in Table 9. Table 10 compares the proposed algorithm’s global Shannon entropy with other encryption methods in the literature. The calculated entropy values of the proposed algorithm are closer to the ideal entropy and higher than those of the compared methods.

### 4.5. Encryption Quality

Encryption quality is an important factor in testing an image encryption method’s efficiency. To quantify the quality of encryption, different tests are performed on the plaintext images and their respective cipher images, such as mean square error (MSE), PSNR, maximum deviation, irregular deviation, and deviation from the uniform histogram. Each of these tests and their results are discussed in detail below.

#### 4.5.1. Mean Square Error (MSE)

MSE is used to measure the average squared difference between the pixel values of two images. The mathematical expression of the MSE between a plaintext image and cipher image is as follows:

(7) $$MSE=\frac{1}{H\times W}\sum_{i=1}^{H}\sum_{j=1}^{W}{\left[P\left(i,j\right)-C\left(i,j\right)\right]}^{2},$$

where *H* and *W* are the height and width of the image, respectively. P(i,j) and C(i,j) are the pixel values of the plaintext image and the cipher image at position (i,j), respectively. For an algorithm to be considered secure, the MSE should have high values, generally ≥30 [22]. Table 11 lists the MSE values between the plaintext and cipher images for the proposed algorithm. Table 12 compares the proposed algorithm with other image encryption methods in the literature. The comparison results show that images encrypted by the proposed algorithm have better encryption quality with respect to the MSE metric as compared to other encryption methods.

#### 4.5.2. Mean Absolute Error (MAE)

MAE is used to measure the difference between the pixel values of two images. The mathematical expression of MAE between the plaintext image and the cipher image is as follows:

(8) $$MAE=\frac{1}{H\times W}\sum_{i=1}^{H}\sum_{j=1}^{W}\left|P\left(i,j\right)-C\left(i,j\right)\right|,$$

where *H* and *W* denote the image’s height and width, respectively. P(i,j) and C(i,j) denote the pixel values of the plaintext image and the cipher image at position (i,j), respectively. The algorithm is considered to have better encryption quality when the MAE value is large. Table 13 lists the MAE values for the proposed algorithm. Table 14 compares the proposed algorithm with other image encryption methods in the literature. The comparison results show that images encrypted by the proposed algorithm have better encryption quality with respect to the MAE metric than other encryption methods.

#### 4.5.3. Peak Signal-to-Noise Ratio

This metric measures the noise ratio between the plain and cipher images. The mathematical expression of PSNR between the plaintext image and cipher image is as follows:

(9) $$PSNR=20\log_{10}\frac{MAX_{p}}{MSE},$$

where $MAX_{p}$ is the maximum value a pixel could have (i.e., 255 in 8-bit pixels), and MSE is the mean squared error value as calculated in Equation (7). A greater PSNR value indicates better image quality and similarity of the cipher image to the plaintext image, which means that a lower value of PSNR indicates a better encryption quality. Table 15 lists the PSNR values of encrypted images produced by the proposed algorithm, and Table 16 compares the PSNR values of the proposed algorithm to other image encryption methods in related literature. The comparison results show that images encrypted by the proposed algorithm have lower PSNR values and thus better encryption quality than other encryption methods.

#### 4.5.4. Maximum Deviation

This metric is used to measure the deviation between the histograms of plaintext and cipher images. The encryption algorithm is considered to have better encryption quality if the cipher image is highly deviated from the plaintext image [27]. The maximum deviation can be calculated as follows:

(10) d=histogram(|P−C|)

(11) $$D_{max}=\frac{d_{0}+d_{255}}{2}+\sum_{i=1}^{254}d_{i},$$

where $D_{max}$ is the maximum deviation metric, *d* is the histogram of the absolute values of the difference between the plaintext and cipher images, $d_{i}$ is the histogram value of *d* at index *i*, and $d_{0}$ and $d_{255}$ are the histogram values at index 0 and 255, respectively. The maximum deviation for the images Baboon, Cameraman, and Peppers encrypted with the proposed algorithm are compared with other related encryption methods in Table 17. The results of the maximum deviation of the cipher images produced by the proposed algorithm are highly deviated from the original image. However, even though the performance of Arif et al. [7] is better than the proposed algorithm for some encrypted images, the average of the proposed method is still generally better.

#### 4.5.5. Irregular Deviation

This metric measures the deviation of individual pixels of plaintext and cipher images. The encryption algorithm is considered to have better encryption quality if the cipher image has a lower irregular deviation value. Irregular deviation can be calculated as follows:

(12) $$D_{irregular}=\sum_{i=0}^{255}\left[\left|d_{i}-D_{avg}\right|\right],$$

where

(13) $$D_{avg}=\frac{1}{256}\sum_{i=0}^{255}d_{i}$$

In Equations (12) and (13), $d_{i}$ is the histogram value *d* at index *i* (from Equation (10)), and $D_{avg}$ is the average value of the pixels that are deviated at every deviation value. The irregular deviation for the images Baboon, Cameraman, and Peppers encrypted with the proposed algorithm are presented and compared to related methods in Table 18. Results indicate that the proposed algorithm has smaller irregular deviation values for most of the encrypted images compared to other encryption methods. However, the performance of Belazi et al. [28] is better than the proposed algorithm for the image Peppers and on average.

#### 4.5.6. Deviation from Uniform Histogram

This metric measures the deviation of the cipher image from a uniform histogram distribution. The better the encryption algorithm, the closer the histogram of the cipher image is to the uniform histogram. Deviation from uniform histogram can be calculated as follows:

(14) $$D_{histogram}=\frac{\sum_{i=0}^{255}\left|H_{C_{i}}-H_{U_{i}}\right|}{H\times W},$$

$$H_{U_{i}}=\left\{\begin{array}{cc} \frac{H\times W}{256} & \mathrm{if}\;0\;\leq i\leq 255\\ 0 & \mathrm{if}\;0\;>i>255 \end{array}\right.$$

where *H* and *W* are the height and width of the image, respectively, and $H_{U_{i}}$ is the uniform histogram value of the *i*th index. Values of $H_{U_{i}}$ are counted only if the pixel value is between 0 to 255, otherwise, the value is counted as zero. $H_{C_{i}}$ is the cipher image histogram value of the *i*th index. The deviations from uniform histogram for the images Baboon, Cameraman, and Peppers encrypted with the proposed algorithm are presented in Table 19 and compared to other encryption methods. The results indicate that the deviations from uniform histogram for all of the cipher images produced by the proposed algorithm are lower than the compared methods.

### 4.6. Resistance to Differential Attacks

Attackers utilize differential attacks (also known as chosen plaintext attacks) to guess the relation between the plaintext image and its cipher image to break the encryption algorithm [4]. In this method, the attacker encrypts two plaintext images with a 1-bit difference and compares the resulting cipher images. For an encryption algorithm to be considered secure, a pixel change in the plaintext image should yield a completely different cipher image. To evaluate an algorithm’s resistance to differential attacks, a set of commonly used tests are performed. These tests are: the avalanche effect, number of pixels change rate (NPCR), and the unified averaged changing intensity (UACI). Each of these tests and their results are discussed in detail below.

#### 4.6.1. Avalanche Effect

**Figure 7:**
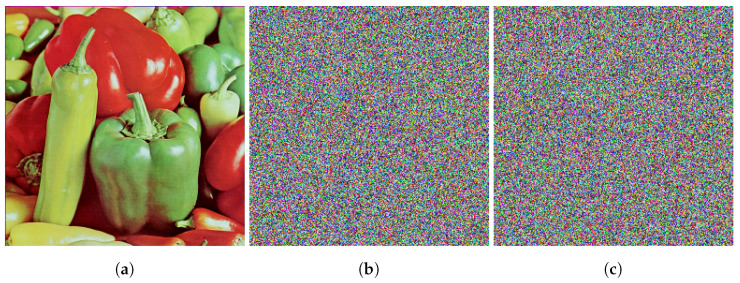
Avalanche effect illustrated in two images encrypted from the same plaintext image with a change of 1 bit: (**a**) Peppers, (**b**) Peppers cipher, (**c**) Peppers cipher where plaintext peppers have 1-bit change.

In cryptography, the property where a slight change in the input changes the output significantly is known as the avalanche effect. The MSE metric can be used to test the avalanche effect with a slight change in the parameters of Equation (7) to calculate the average squared difference between the pixel values of two cipher images encrypted from the same plaintext image with a change of 1-bit instead of between the plaintext and cipher image. The avalanche effect MSE can be calculated as follows:

(15) $$MSE_{av}=\frac{1}{H\times W}\sum_{i=1}^{H}\sum_{j=1}^{W}{\left[C_{1}\left(i,j\right)-C_{2}\left(i,j\right)\right]}^{2},$$

where *H* and *W* are the height and width of the images, respectively, and $MSE_{av}$ denotes avalanche effect MSE. $C_{1}$ and $C_{2}$ are two cipher images encrypted from the same plaintext image with a change of 1 bit. $C_{1}\left(i,j\right)$ and $C_{2}\left(i,j\right)$ are the pixel values of the cipher images 1 and 2 at index (i,j), respectively. Figure 7 depicts the plaintext image Peppers and the resulting ciphers with a change of 1 bit. If a 1-bit change in the plaintext image causes a change of more than 50% in the cipher image, then the algorithm is safe against differential attacks [30]. Table 20 depicts the $MSE_{av}$ for various images encrypted by the proposed algorithm.

#### 4.6.2. Number of Pixels Change Rate (NPCR)

This metric sums the differences between two cipher images that are encrypted from the same plaintext image, but with a change of 1 bit in the plaintext. This metric is useful to test an encryption algorithm’s resilience against differential attacks. NPCR can be calculated as:

(16) $$NPCR=\sum_{i=1}^{H}\sum_{j=1}^{W}D\left(i,j\right),$$

where

$$D\left(i,j\right)=\left\{\begin{array}{cc} 0 & \mathrm{if}\;C_{1}\left(i,j\right)=C_{2}\left(i,j\right)\\ 1 & \mathrm{if}\;C_{1}\left(i,j\right)\neq C_{2}\left(i,j\right) \end{array}\right.$$

where *H* and *W* are the height and width of the image, respectively, and $C_{1}$ and $C_{2}$ are two cipher images encrypted from the same plaintext image with a change of 1 bit. $C_{1}\left(i,j\right)$ and $C_{2}\left(i,j\right)$ are the pixel values of the cipher images 1 and 2 at position (i,j), respectively. D(i,j) has a value of 0 if there is no difference in the pixel values of $C_{1}$ and $C_{2}$ at position (i,j), and a value of 1 if the pixel values of $C_{1}$ and $C_{2}$ at position (i,j) are different.

Table 20 depicts the NPCR values for various images (Baboon, Cameraman, Male, Peppers, and Sailboat) encrypted by the proposed algorithm. The higher the NPCR value, the higher an algorithm’s responsiveness to plaintext change is. The ideal average value of NPCR is ≥99.6094 [31]. Table 21 compares the average NPCR values of the proposed algorithm with other encryption algorithms discussed in the literature. The results show that the proposed algorithm has a higher average NPCR value compared to other algorithm and the ideal value for NPCR. However, the performance of the algorithm proposed by Alanezi et al. [6] is slightly better than the proposed algorithm with respect to the NPCR metric.

#### 4.6.3. Unified Average Changing Intensity (UACI)

In image encryption, UACI is used to measure the change in the average intensity between two images encrypted from the same plaintext image with a change of 1 bit. UACI can be calculated as follows:

(17) $$UACI=\frac{1}{H\times W}\left[\frac{\sum_{i=1}^{H}\sum_{j=1}^{W}\left|C_{1}\left(i,j\right)-C_{2}\left(i,j\right)\right|}{2^{b}-1}\right]\times 100\%,$$

where *H* and *W* are the height and width of the image, respectively. $C_{1}\left(i,j\right)$ and $C_{2}\left(i,j\right)$ are the pixel values of the cipher images 1 and 2 at position (i,j), respectively, and *b* is the number of bits that represent the image pixel.

Table 20 depicts the UACI values for various images (Baboon, Cameraman, Male, Peppers, and Sailboat) encrypted by the proposed algorithm. The higher the UACI values, the higher the algorithm’s responsiveness to plaintext change is. The ideal average value of UACI is ≥33.4635 [31]. Table 21 compares the UACI of the proposed algorithm with other encryption algorithms. The results shows that the proposed algorithm has a higher average UACI value compared to other algorithms and the ideal value. However, the performance of the encryption algorithm proposed by Alanezi et al. [6] is slightly better than the proposed algorithm with respect to the UACI metric. Table 22 compares the correlation coefficient, entropy, PSNR, NPCR, UACI, and encryption time of the proposed algorithm with other encryption algorithms.

### 4.7. Resistance to Noise and Data Loss Attacks

**Figure 8:**
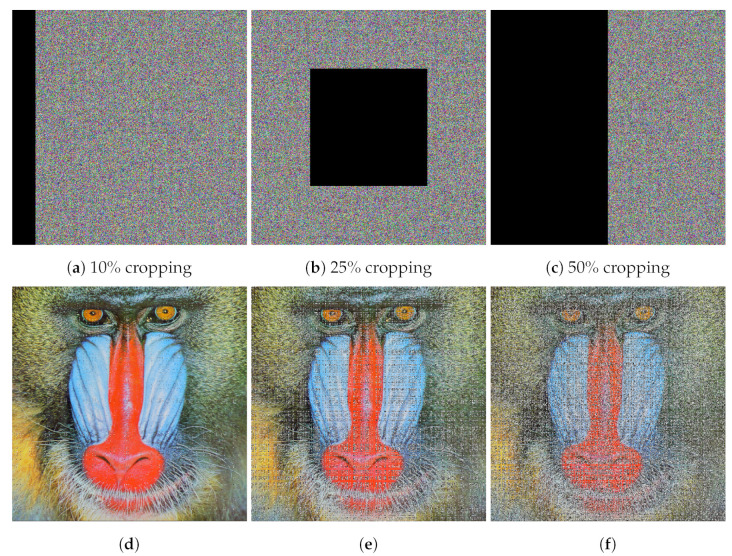
Results of data loss (occlusion) attacks on the color image Baboon (512×512): (**a**–**c**) different severities of occlusion attacks, (**d**–**f**) corresponding decrypted images.

**Figure 9:**
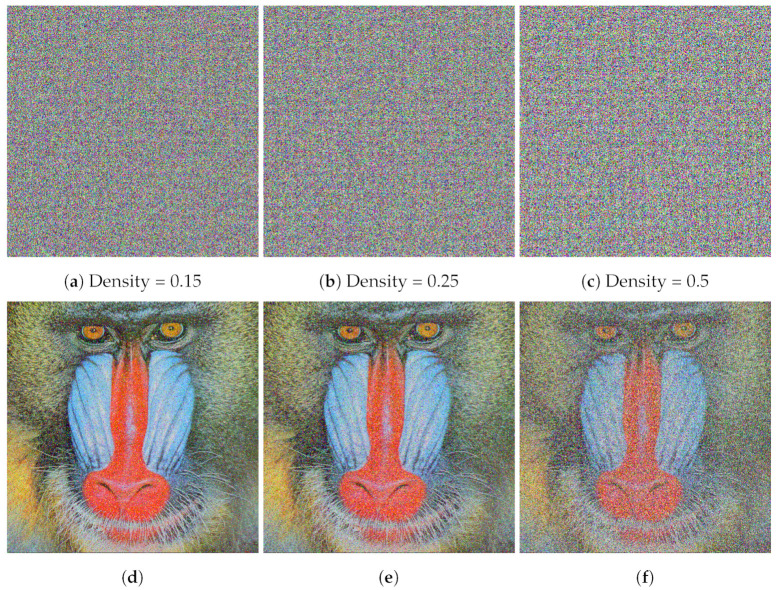
Results of salt and pepper noise attack on the color image Baboon (512×512): (**a**–**c**) different noise densities introduced, (**d**–**f**) corresponding decrypted images.

Digital images transferred over a network are prone to noise and data loss that could affect the encrypted image. Data loss attacks are sometimes also referred to as occlusion attacks. An *occlusion attack* is a cyberattack where an adversary with the capability to alter cipher image data can crop part of the image out to thwart decryption or to render the decrypted image useless. An image encryption algorithm should be robust against noise and data loss attacks and should be able to decrypt the affected cipher image. We have evaluated the robustness of the proposed algorithm against data loss attacks by cropping parts of the cipher image. To measure the robustness against noise, we have added salt and pepper noise in the cipher image. Figure 8 depicts data loss (occlusion) attacks on a cipher image with different severities (10% cropping, 25% cropping, and 50% cropping). Figure 8 also illustrates that the plaintext image is successfully recovered by decrypting the affected cipher image with cropping up to 50% using our proposed encryption/decryption algorithm. Figure 9 shows cipher images in which salt and pepper noise has been introduced with different densities (0.15, 0.25, and 0.5). We note that a noise density of 0.15 affects approximately 15% of pixels, a noise density of 0.25 affects approximately 25% of pixels, and a noise density of 0.5 affects approximately 50% of pixels in the image. Figure 9 shows that our proposed algorithm is able to decrypt cipher images affected by salt and pepper noise density up to 0.5.

### 4.8. Homogeneity

Homogeneity analysis quantifies the closeness of the distribution of elements in the gray-level co-occurrence matrix (GLCM) to the GLCM diagonal. The GLCM calculates the frequency of a pixel with gray-level value *i* occurring horizontally adjacent to a pixel with the gray-level value *j* [33]. The GLCM is used for feature and texture extraction in image processing. The range of homogeneity is [0, 1], and the algorithm is considered more efficient if the homogeneity value is lower. Homogeneous parts of an image reveal repetitive structures and compromise the image encryption [34]. The homogeneity can be calculated as follows:

(18) $$Homogeneity=\sum_{i,j}\frac{p(i,j)}{1+|i-j|},$$

where *i* and *j* are two gray-level values occurring horizontally adjacent to each other, and p(i,j) is the value of the element at position (i,j) in the normalized GLCM. In the normalized GLCM, the sum of its elements is equal to 1, and each element p(i,j) in the normalized GLCM is the joint probability occurrence of pixel pairs with a defined spatial relationship having gray level values *i* and *j* in the image. Table 23 lists the results of the homogeneity analysis of the plaintext and cipher images. These results show that the proposed algorithm produces low values of homogeneity. Table 24 compares the homogeneity results of the proposed algorithm and other image encryption methods. Results in Table 24 indicate that our proposed algorithm produces the lowest homogeneity values when compared to other encryption methods.

### 4.9. Contrast

Contrast analysis measures the intensity contrast between a pixel and its neighbor over the whole image. The algorithm is considered more secure if the cipher image has high values of contrast. A higher level of contrast suggests a higher level of randomness in the cipher image. The contrast can be calculated as follows:

(19) $$Contrast=\sum_{i,j}{\left|i-j\right|}^{2}p\left(i,j\right),$$

where *i* and *j* are two gray-level values occurring horizontally adjacent to each other. Table 25 shows the results of the contrast analysis of the plaintext and cipher images for the proposed algorithm. These results show that the proposed algorithm produces high contrast levels in the encrypted image. Table 24 compares the contrast value produced by the proposed algorithm versus other image encryption methods. The results in Table 24 indicate that our proposed algorithm produces higher contrast value compared to other encryption methods.

### 4.10. Energy

Energy (also known as uniformity) measures the sum of squared elements in the GLCM. The range of energy is [0, 1], and the algorithm is considered more secure if the cipher image has a lower energy value. The energy can be calculated as follows:

(20) $$Energy=\sum_{i,j}p{\left(i,j\right)}^{2},$$

where *i* and *j* are two gray-level values occurring horizontally adjacent to each other. Table 26 shows the results of the energy analysis of plaintext and cipher images for our proposed algorithm. Table 24 compares the results of the energy analysis of cipher images produced by the proposed algorithm versus other encryption methods. The results in Table 24 show that the energy value of the cipher image from our proposed algorithm is among the algorithms with the lowest energy values.

### 4.11. NIST SP 800-22 Test

We have utilized the NIST SP 800-22 test suite [38] (version 2.1.2) to check the randomness characteristics and measure the strength property of a random sequence where the sequence should be unpredictable. The test suite performs 15 statistical tests, and each test generates a *p*-value in the range [0, 1]. If the *p*-value of a test is greater than the threshold value of μ = 0.01, then the sequence is considered to be random and passes the test. Table 27 presents the results of the NIST SP 800-22 tests for the encrypted Baboon image, which is encrypted through our proposed algorithm. The results in Table 27 indicate that the sequence of the cipher image Baboon passes all NIST SP 800-22 tests.

### 4.12. Key Space and Sensitivity Analyses

The key space is the set of all possible keys that could be used by a cryptosystem. To provide sufficient security against brute force attacks, key space for an encryption algorithm should be larger than $2^{100}$ [39]. The proposed algorithm relies on a 256-bit hash generated from the plaintext image using SHA-2 256, a 256-bit pre-shared secret key, and a 256-bit IV, which are XORed together to produce a derived security key with a length of 256 bits, which is supplied as an input parameter to the logistic map (Algorithm 1). Thus, the key space for the proposed algorithm is $2^{256}$, which is large enough to withstand brute force attacks.

**Figure 10:**
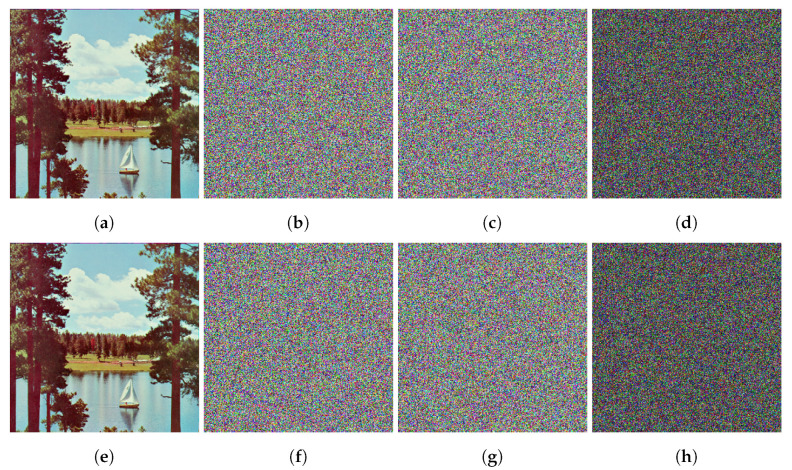
Results of key sensitivity tests on the color image Sailboat (512×512): (**a**) plaintext image, (**b**) $C_{1}$ encrypted using key $K_{1}$, (**c**) $C_{2}$ encrypted using key $K_{2}$, (**d**) difference of images: |(**b**) − (**c**)|, (**e**) $C_{1}$ decrypted using key $K_{1}$, (**f**) $C_{1}$ decrypted using key $K_{2}$, (**g**) $C_{2}$ decrypted using key $K_{1}$, (**h**) difference of images: |(**f**) − (**g**)|.

Key sensitivity analyzes an encryption algorithm’s sensitivity to slight changes in the encryption key. A secure encryption algorithm should produce a completely different cipher image when a plaintext image is encrypted with two keys that differ only in a single bit. Furthermore, the algorithm should not be able to recover the plaintext image from a cipher image with the key changed by 1 bit. To evaluate the proposed algorithm’s key sensitivity, we have encrypted the color image Sailboat (512×512) twice using two keys, $K_{1}$ and $K_{2}$, which differ only in 1 bit. The proposed algorithm produces two completely different cipher images, $C_{1}$ and $C_{2}$. For the decryption process, the plaintext image is not recoverable when trying to decrypt $C_{1}$ using $K_{2}$, or $C_{2}$ using $K_{1}$. To quantify the key sensitivity analysis, we have calculated the NPCR (Equation (16)) and UACI (Equation (17)) values for the cipher images. The NPCR value of encrypted images $C_{1}$ and $C_{2}$ when encrypted with the 1-bit-changed key is 99.6465, and the UACI value is 33.4942, which are very close to the ideal values for both metrics. Figure 10 illustrates the key sensitivity test for the proposed algorithm. The results show that when encrypting the same plaintext image with keys that differ only by 1 bit, the algorithm produces completely different cipher images. Furthermore, results reveal that the plaintext image is not recoverable when using the 1-bit-changed key. These results verify that the proposed algorithm has high sensitivity to minor changes in the secret key.

## 5. Conclusions

In this paper, an efficient and secure lightweight chaos-based image encryption algorithm is proposed. Our proposed algorithm’s encryption and decryption processes use logistic maps to produce the chaotic permutation and XOR keys, and AES S-Box to substitute the values of the pixels of the image to further randomize the produced cipher image. We have analyzed and compared our proposed image encryption algorithm with other encryption methods using a variety of security, quality, and efficiency metrics. Experimental results reveal that the proposed algorithm has the lowest execution time compared to other encryption methods. Results show that the proposed algorithm is up to 15.33× faster compared to other contemporary encryption methods. Experimental results further show that the proposed algorithm produces promising results in correlation coefficient, histogram, chi-square, entropy, homogeneity, contrast, and energy analysis. Encryption quality tests, namely mean square error, mean absolute error, peak signal-to-noise ratio, maximum deviation, irregular deviation, and deviation from the uniform histogram, demonstrate that the proposed algorithm yields better quality encryption compared to other image encryption methods. The proposed algorithm also shows high resistance against differential attack when tested for avalanche effect, NPCR, and UACI. Furthermore, the proposed algorithm provides high resistance against noise and data loss attacks. Results reveal that our proposed encryption/decryption algorithm is able to recover the plaintext image when decrypting the cipher image with up to 50% data loss and up to 50% of pixels affected by noise. We have used the NIST SP 800-22 test suite to test the randomness characteristics of the cipher image produced by the proposed algorithm, and the results show that the cipher image encrypted by our proposed algorithm passes all the tests in the NIST SP 800-22 test suite.

The proposed algorithm uses logistic maps for their minimal complexity and fast computation speed, but the logistic maps have a low key space. In our future work, we plan to explore different chaotic maps that could be utilized by our proposed image encryption algorithm to generate pseudorandom numbers that could provide higher security, an even distribution of pseudorandom numbers, and a larger key space compared to the logistic map.

## Figures and Tables

**Table 1:** An example of the generation of Matrix 1 ($M_{1}$) from the logistic map.

22	140	246	206	238
76	186	252	20	134
78	251	21	164	214
82	68	86	180	12
166	226	136	215	144

**Table 2:** Average encryption time (in seconds) of the proposed algorithm for different image dimensions.

Image Size	Color	Time (s)
256×256	Gray	0.0235
256×256	Color	0.0287
512×512	Gray	0.0915
512×512	Color	0.1056
1024×1024	Gray	0.4124
1024×1024	Color	0.4386

**Table 3:** Average encryption time (in seconds) of several color and gray scale 512×512 images for the proposed algorithm compared to different related methods.

	Proposed	[5]	[6]	[7]	[9]
Time (s)	**0.09155**	0.2338	0.3033	1.28	1.489

**Table 4:** Encryption time (in seconds) for the proposed algorithm compared to different related methods scaled to 3.5 GHz CPU.

	Proposed	[6]	[9]	[17]
Time (s)	**0.09155**	0.2166	1.4039	0.2102
Speed up	-	2.36	15.33	2.30

**Table 5:** Vertical, horizontal, and diagonal correlation coefficients of adjacent pixels of encrypted images.

		Correlation Coefficient		
Image	Color and Size	Vertical	Horizontal	Diagonal
Baboon	512×512 Color	−0.0001	0.0006	−0.0021
Cameraman	512×512 Gray	−0.0039	−0.0003	0.0037
Male	1024×1024 Gray	0.0004	0.0017	−0.0009
Peppers	256×256 Color	−0.0003	0.0009	−0.0006
Sailboat	512×512 Color	−0.0012	0.0016	0.0001
Average		0.0005	0.0004	−0.0004

**Table 6:** Vertical, horizontal, and diagonal correlation coefficients of encrypted Baboon image of the proposed algorithm compared to different encryption methods.

	Correlation Coefficient		
	Vertical	Horizontal	Diagonal
Proposed	**−0.0001**	0.0006	−0.0021
[5]	−0.0086	0.0023	0.0402
[6]	**−0.0001**	**−0.0002**	**0.0011**
[7]	−0.0036	−0.0019	−0.0033
[9]	−0.0004	0.0007	0.0029

**Table 7:** Vertical, horizontal, and diagonal correlation coefficients of plaintext and cipher images.

		Correlation Coefficient		
Image	Color and Size	Vertical	Horizontal	Diagonal
Baboon	512×512 Color	−0.0029	−0.0550	−0.0005
Cameraman	512×512 Gray	−0.0912	−0.0353	0.0035
Male	1024×1024 Gray	0.0164	−0.0226	−0.0007
Peppers	256×256 Color	−0.0025	0.0023	0.0002
Sailboat	512×512 Color	−0.1277	0.0265	0.0012
Average		−0.0372	−0.0061	0.0003

**Table 8:** Chi-square test values for plaintext images and cipher images encrypted by our proposed algorithm.

		Chi-Square	
Image	Size and Color	Plaintext Image	Cipher Image
Baboon	512×512 color	10,429,131.335	258.671
Cameraman	512×512 gray	2,193,251.085	247.173
Male	1024×1024 gray	26,095,050.882	299.851
Peppers	256×256 color	557,983.062	286.742
Sailboat	512×512 color	424,683.429	273.918
Average			273.271

**Table 9:** Global and local Shannon entropy values of images encrypted by our proposed algorithm.

		Entropy	
Image	Size and Color	Global	Local
Baboon	512×512 Color	7.9997	7.8979
Cameraman	512×512 Gray	7.9991	7.8978
Male	1024×1024 Gray	7.9996	7.9026
Peppers	256×256 Color	7.9990	7.8883
Sailboat	512×512 Color	7.9988	7.8880
Average		7.9992	7.8949

**Table 10:** Global entropy of cipher image Baboon of the proposed algorithm compared with different encryption algorithms.

	Proposed	[5]	[6]	[7]	[9]
Entropy	**7.9997**	7.9024	7.9991	7.9992	7.9971

**Table 11:** MSE values of images encrypted by the proposed algorithm.

Image	Size and Color	MSE
Baboon	512×512 color	46.0304
Cameraman	512×512 gray	49.8847
Male	1024×1024 gray	53.8392
Peppers	256×256 color	23.5767
Sailboat	512×512 color	53.6684
Average		42.3794

**Table 12:** Mean square error (MSE) for images encrypted by the proposed algorithm compared to related methods.

	Proposed	[7]	[22]	[23]
MSE	**42.3794**	39.6794	33.4275	40.3295

**Table 13:** MAE values of images encrypted by the proposed algorithm.

Image	Size and Color	MAE
Baboon	512×512 color	89.69
Cameraman	512×512 gray	96.56
Male	1024×1024 gray	97.55
Peppers	256×256 color	86.47
Sailboat	512×512 color	87.64
Average		91.58

**Table 14:** Average MAE values for images encrypted by the proposed algorithm compared to related methods.

	Proposed	[24]	[25]	[26]
MAE	**91.58**	79.57	78.10	90

**Table 15:** Peak signal-to-noise ratio (PSNR) of images encrypted by the proposed algorithm.

Image	Size and Color	PSNR
Baboon	512×512 color	8.7880
Cameraman	512×512 gray	8.4124
Male	1024×1024 gray	8.0008
Peppers	256×256 color	8.6210
Sailboat	512×512 color	8.7439
Average		8.4458

**Table 16:** Peak signal-to-noise ratio (PSNR) of the images encrypted by the proposed algorithm compared to other encryption methods.

	Proposed	[6]	[7]	[24]	[17]
PSNR	**8.4458**	8.6449	9.5424	9.0996	9.7936

**Table 17:** Maximum deviation results for proposed algorithm compared to related methods.

Image	Proposed	[7]	[28]
Baboon	**363,121**	199,158	-
Cameraman	64,382	**64,998**	18,007
Peppers	**209,618**	146,408	22,935
Average	**167,482**	102,022	20,109

**Table 18:** Irregular deviation results for the proposed algorithm compared to other encryption methods.

Image	Proposed	[7]	[28]
Baboon	**59,921**	80,203	-
Cameraman	**32,165**	32,706	39,244
Peppers	76,075	84,465	**35,088**
Average	91,460	129,253	**40,739**

**Table 19:** Deviation from uniform histogram for the proposed algorithm compared with other encryption methods.

Image	Proposed	[28]	[29]
Baboon	**0.0256**	-	0.0496
Cameraman	**0.0305**	0.0942	0.0502
Peppers	**0.0315**	0.0917	-
Average	**0.0382**	0.0534	0.04965

**Table 20:** Avalanche effect ${\mathrm{MSE}}_{av}$, NPCR, and UACI values of images encrypted by the proposed algorithm.

Image	Size and Color	MSE${}_{\mathrm{av}}$	NPCR	UACI
Baboon	512×512 color	57.3895	99.6125	33.5531
Cameraman	512×512 gray	57.3470	99.6368	33.3911
Male	1024×1024 gray	57.2375	99.6146	33.4947
Peppers	256×256 color	57.4122	99.6218	33.4862
Sailboat	512×512 color	57.4562	99.6176	33.4993
Average		57.3533	99.6153	33.4718

**Table 21:** NPCR and UACI of the proposed algorithm compared with other encryption methods.

	NPCR	UACI
Proposed	99.6153	33.4718
[5]	99.5893	33.3730
[6]	**99.6239**	**33.5615**
[7]	99.6059	33.4375
[9]	99.5743	33.3941
[32]	99.6146	33.4501

**Table 22:** Vertical, horizontal, and diagonal correlation coefficient, PSNR, NPCR, UACI, and the encryption time of the proposed algorithm compared to other encryption methods.

Algorithm		Proposed	[5]	[6]	[7]	[9]	[24]	[17]
Correlation coefficient	Vertical	0.0005	−0.0086	**−0.0001**	−0.0036	−0.0004	−0.0357	-
	Horizontal	0.0004	0.0023	**−0.0002**	−0.0019	0.0007	−0.0357	-
	Diagonal	**−0.0004**	0.04024	0.0011	−0.0033	0.0029	−0.0223	-
Entropy		**7.9997**	7.9024	7.9991	7.9992	7.9971	7.9985	7.9969
PSNR		**8.4458**	-	8.6449	9.5424	-	9.0996	9.7936
NPCR		99.6153	99.5893	**99.6239**	99.6059	99.5743	99.6269	99.6100
UACI		33.4718	33.3730	**33.5615**	33.4375	33.4561	33.3514	33.5200
Encryption time	(512×512)	**0.09155 s**	0.2332 s	0.3033 s	1.28 s	1.243 s	22.43 s	0.736

**Table 23:** Homogeneity analysis for plaintext and cipher images.

Image	Size and Color	Plaintext Image	Cipher Image
Baboon	512×512 color	0.7652	0.3893
Cameraman	512×512 gray	0.8979	0.3898
Male	1024×1024 gray	0.8475	0.3897
Peppers	256×256 color	0.9353	0.3896
Sailboat	512×512 color	0.8664	0.3897
Average			0.3897

**Table 24:** Comparison of homogeneity, contrast, and energy analysis of cipher image of the proposed algorithm and related methods.

	Image Color	Homogeneity	Contrast	Energy
Proposed	Color	**0.3837**	**10.5081**	0.01563
[24]	Color	0.3895	10.5079	0.01562
[35]	Gray scale	0.4208	8.3301	0.01760
[36]	Color	0.4110	8.6448	**0.01561**
[37]	Gray scale	0.3916	10.4252	0.01563

**Table 25:** Contrast analysis for plaintext and cipher images.

Image	Size and Color	Plaintext Image	Cipher Image
Baboon	512×512 color	0.7425	10.5152
Cameraman	512×512 gray	0.1978	10.4670
Male	1024×1024 gray	0.2527	10.4918
Peppers	256×256 color	0.2665	10.4893
Sailboat	512×512 color	0.4140	10.4868
Average			10.5081

**Table 26:** Energy analysis for plaintext and cipher images.

Image	Size and Color	Plaintext Image	Cipher Image
Baboon	512×512 color	0.0639	0.01564
Cameraman	512×512 gray	0.1939	0.01565
Male	1024×1024 gray	0.1199	0.01563
Peppers	256×256 color	0.1437	0.01563
Sailboat	256×256 color	0.1155	0.01563
Average			0.01563

**Table 27:** Results of NIST SP 800-22 tests for the encrypted Baboon image.

Test		*p*-Value	Passed
Frequency		0.213309	✓
Block Frequency		0.350485	✓
Cumulative Sums	Reverse	0.350485	✓
Cumulative Sums	Forward	0.534146	✓
Runs		0.066882	✓
Longest Run of Ones		0.534146	✓
Rank		0.534146	✓
FFT		0.739918	✓
Non-Overlapping Template		0.911413	✓
Overlapping Template		0.911413	✓
Universal		0.122325	✓
Approximate Entropy		0.213309	✓
Random Excursions		0.742591	✓
Random Excursions Variant		0.728588	✓
Serial	Test 1	0.739918	✓
Serial	Test 2	0.213309	✓
Linear Complexity		0.350485	✓

## Data Availability

The data presented in this study are available on request from the corresponding author on reasonable request. The data are not publicly available due to proprietary reasons.
